# Patch Based Grid Artifact Suppressing in Digital Mammography

**DOI:** 10.1155/2018/9727259

**Published:** 2018-08-12

**Authors:** Qingqing Ling, Shuyu Wu, Xiaoman Duan, Genggeng Qin, Jianhui Ma, Chaomin Chen, Hongliang Qi, Linghong Zhou, Yuan Xu

**Affiliations:** ^1^School of Biomedical Engineering, Southern Medical University, Guangzhou 510515, China; ^2^Department of Radiology, Nanfang Hospital, Southern Medical University, Guangzhou 510515, China

## Abstract

The mammography is the first choice of breast cancer screening, which has proven to be the most effective screening method. An antiscatter grid is usually employed to enhance the contrast of image by absorbing unexpected scattered signals. However, the grid pattern casts shadows and grid artifacts, which severely degrade the image quality. To solve the problem, we propose the patch based frequency signal filtering for fast grid artifacts suppressing. As opposed to whole image processing synchronously, the proposed method divides image into a number of blocks for tuning filter simultaneously, which reduces the frequency interference among image blocks and saves computation time by multithread processing. Moreover, for mitigating grid artifacts more precisely, characteristic peak detection is employed in each block automatically, which can accurately identify the location of the antiscatter grid and its motion pattern. Qualitative and quantitative studies were performed on simulation and real machine data to validate the proposed method. The results show great potential for fast suppressing grid artifacts and generating high quality of digital mammography.

## 1. Introduction

 Breast cancer ranks as the first leading cancer in women all over the world [1]. According to the Global Burden of Disease Report [2, 3], the newly increased incidence and death tolls of breast cancer are on the rise around the world, which has accounted for one-quarter of the total new cases of women. The early screening and diagnosis of breast cancer are helpful to improve the survival fraction and quality of life [4]. Imaging examination is one of the most significant measurements in the breast cancer screening [5], which includes B-ultrasound, CT, MRI, and X-ray mammography [6–10]. The X-ray mammography is the first choice of breast cancer screening, which has proven to be the most effective screening method by World Health Organization [1]. When X-rays pass through the breast tissue, the irregular direction scattered signals are generated because of the Compton effect and Rayleigh scattering [11]. An antiscatter grid between the patient and the image detector is usually employed to enhance the contrast of image by absorbing unexpected scattered signals [12], and the grid consists of alternating transmitting material and absorbing material of X-ray. However, the grid pattern casts shadows on the image detector and produces grid artifacts in the acquired X-ray image due to the existence of absorbing material [13, 14]. The artifacts severely degrade the image quality. Hence, grid artifact suppressing is the prerequisite and foundation in digital mammography.

To address the issue, various approaches have been proposed. The grid artifacts suppressing methods are classified as hardware methods and image processing methods. The first category utilizes grid techniques to depress artifacts. For instance, moving grids is frequently used to mitigate grid artifacts [12] with oscillating and reciprocating as basic moving mechanism. However, this technique is employed with extra complexity. By analyzing grid movements, Bednarek et al. [15] found that small grid movements could reduce artifacts under the conditions of the synchronization between linear grid motion and exposure time. Gauntt and Barnes introduced a comprehensive theory on the antiscatter grids [16] and also proposed an artifact suppression technique [17], and in this technique the grid moved at a modest velocity when the X-ray exposure waveform had a soft start and stop. Those algorithms may be complicated due to the dependence of gird movement. Moreover, a high line density of the grid is necessary for obtaining more perfect image quality.

Image processing methods mainly depend on digital image processing techniques [18] rather than grid movement. For example, Wang and Huang [19] proposed a mathematical method to remove aliasing artifacts based on studying the amplitudes and the frequencies of artifacts and converted a film into digital form. Barski and Wang [20] proposed a method for grid artifacts reducing based on one-dimensional discrete Fourier transform and one-dimension frequency filtering by structuring blur kernels. Moreover, the one-dimension notch filter was also used by Belykh and Cornelius [21]. Unfortunately, the ripple artifacts were produced in the image. Different from the one-dimensional method based on frequency domain, Sasada et al. [22] proposed two-dimensional filtering based on the wavelet domain to alleviate grid artifacts. In another study by Lin et al. [23], Gaussian band-stop filters were conducted to reduce artifacts by determining the artifacts frequency. In addition, Zhang et al. [24] constructed an adaptive frequency filter by determining stripe frequency band and frequency distribution function to remove the artifacts. This method is easy to implement, but it was only tested on the infrared image. In order to minimize the damage from grid artifact reduction, Kim and Lee first analyzed grid artifacts with multiplicative model and rotated stationary grids and then removed grid artifacts by constructing the homomorphic filtering consisting of band-stop filters and one-dimensional low-pass filters for searching the optimal grid frequencies and angles [25–28]. Tang et al. decomposed the image into several subimages using a multiscale two-dimensional discrete wavelet transform and the remove gridline signals by an automatic Gaussian band-stop filter [29]. There is higher accuracy of recognizing grid frequency in the method; however, this algorithm may involve fairly long computation time for searching the accurate grid frequencies and angles.

To address the above issues, we propose a patch based method for fast frequency signal filtering and grid artifacts suppressing in digital mammography. As opposed to whole image processing synchronously, the proposed method divides the image into a number of blocks processing simultaneously, which reduces the frequency interference among image blocks as well as saving computation time because of using multithread processing. Moreover, in order to alleviate grid artifacts more precisely, characteristic peak detection is employed in each block automatically, which can accurately identify the position of the antiscatter grid and its motion pattern.

The remaining part of this paper is organized as follows.  Section 2 describes the workflow and each key step in detail.  Section 3 focuses on the implementations to validate the proposed method. Experimental results are shown by simulation study and real digital mammography machine. In Section 4, a few related issues are discussed. Conclusions are given in Section 5.

## 2. Materials and Methods

**Figure 1:**
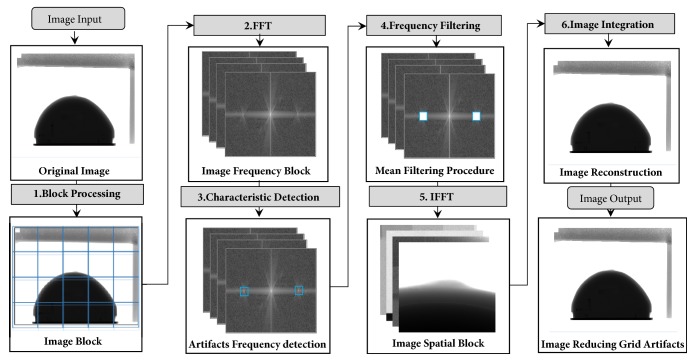
Workflow of the integrated method.

Figure 1 shows a workflow of the proposed method, which consists of six steps. In step 1, the original mammogram is divided into several blocks. In step 2, in spatial domain, two-dimensional FFT is utilized to acquire frequency data of each block. In step 3, in frequency domain, the characteristic frequency detection of grid artifacts is implemented automatically in each frequency block. In step 4, the frequency filtering of characteristic peak is realized by using an improved mean filter. In step 5, the spatial image blocks are obtained by IFFT. Finally, we integrate the spatial image blocks by the inverse operation in step 1. By the above-mentioned steps, the corrected image of the grid artifacts suppressing is realized. The core of the proposed method is in step 1, step 3, and step 4. These steps in the workflow will be detailed and presented in the rest of the section.

### 2.1. Patch Based Deconstruction and Transformation

In this section, image block processing is introduced in detail, which is one of key steps of the proposed method. For a given image* f *(*x*,* y*), block processing can be expressed as

(1) $$\begin{array}{c} f_{b}\left(\;x,y\;\right)={\left.\;f\left(\;x,y\;\right)\;\right|}_{x\in \left[x_{b},x_{b}+X\right],y\in \left[y_{b},y_{b}+Y\right]} \end{array}$$

where (*x*_*b*_, *y*_*b*_) is the top left pixel coordinate of the image block, and* X×Y* is size of the image block.

There are a few points we would like to mention when the patch is applied in image deconstruction. First, image patch processing can decrease the frequency interference between different blocks. In addition, multithread parallel processing can reduce time overload and improve the efficiency of Central Processing Unit (CPU).

The image block is decomposed into sine and cosine components by the FFT. For an image block *f*_*b*_(*x*,* y*) with size of* X×Y*, its expression of the two-dimensional FFT is as follows:

(2) $$\begin{array}{c} F\left(\;u,v\;\right)=\frac{1}{XY}\overset{X-1}{\underset{x=0}{\sum }}\overset{Y-1}{\underset{y=0}{\sum }}f_{b}\left(\;x,y\;\right)e^{-2\pi j\left(ux/X+vy/Y\right)} \end{array}$$

where* F*(*u*,* v*) is the frequency domain data,* u*= 0,1⋯,*X*-1, and* v*= 0,1,⋯,* Y*-1.

### 2.2. Characteristic Peak Detection

**Figure 2:**
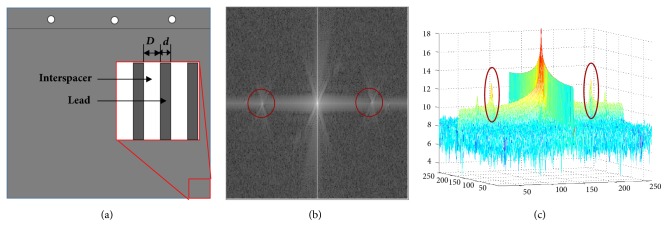
(a) Internal structure of antiscatter grid; (b) block image in the frequency domain; (c) 3D description of frequency components.

In the spatial domain, the grid artifacts can be considered as periodic streak artifacts. So they are expressed as symmetrical signals in the frequency domain [30] as shown in Figures 2(b) and 2(c). The frequency signals of grid artifacts are mainly in the red circles.

As mentioned previously, in order to remove grid artifacts precisely, the characteristic frequency detection of grid signals is conducted without manual intervention. According to sampling theorem and the FFT [31], the characteristic peak range of periodic signals in the frequency domain is defined as

(3) $$\begin{array}{c} N_{f}=\left[\;\varsigma ,\frac{\left(1/SR_{p}\right)\times ld\times Dim}{10}+\sigma \;\right] \end{array}$$

where* ς *and* σ* are length and width of detection range,* Dim *is the image resolution,* SR*_*p*_ is the image block resolution, and* ld *is the grid density.* SR*_*p*_ and* ld *are defined as

(4) $$\begin{array}{c} SR_{p}=\frac{1}{ps} \end{array}$$

where* ps* is the pixel size depending on image detector.

(5) $$\begin{array}{c} ld=\frac{10}{D+d} \end{array}$$

where *D* is the distance between two grids filled with interspacer such as aluminum oxide or plastic fiber in the antiscatter grid,* d* is the width of each grid made by lead [32]. The internal structure of antiscatter grid is shown in Figure 2(a).

After obtaining the range of characteristic peak frequency, we chose the maximum value of* Nf *as filter frequency by the experience and experiments. And* Fmax *is expressed as

(6) $$\begin{array}{c} F_{max}=\max{\left.\;\left(\;F\left(\;u,v\;\right)\;\right)\;\right|}_{\left(u,v\right)\in N_{f}} \end{array}$$

From (3),* Nf *is proportional to image resolution* Dim* and grid density* ld*, so the values of* Dim* and* ld* are lowered while the value of* Nf *is synchronously decreased. The relationship among the three variables indicates that we can obtain high precision of characteristic frequency even with the lower image resolution and common accuracy of grid density.

### 2.3. Frequency Signal Filtering and Reconstruction

For global filtering, the peak attenuation of characteristic frequency happened, which could lead to some loss of image information. Hence, global processing may produce filtering error and have a poor robustness. On the contrary, the block filtering could determine a proper filter size according to the block size and it could reduce the corresponding frequency interference between different blocks. Moreover, if an image block has a filter error, the impact of the error on the whole process could be ignored. Besides, considering that the computational complexities for the global filtering are so serious for the current detector products, we combined block blocking and local filters to improve computation efficiency by using GPU multithread processing. For minimizing the influence of artifacts frequency filtering, we propose an improved filter based on the conventional mean filter [18] to reduce characteristic frequency signals. The expression of filtering procedure is as follows:

(7) Gu,v=meanFu,v∗Hu,vu,v∈D

(8) Hu,v=10⋯0001⋯00⋮⋮1⋮⋮00⋯1000⋯01M×M

where* M×M* is the size of mean filter,* D *is the frequency domain with the size of* M×M, *and its center coordinate is (*u*_1_, *v*_1_) calculated from (6). The fundamental grid frequency indeed contains some harmonic components. Comparing with fundamental components, the harmonics have a higher frequency but a much lower magnitude [23]. And removing harmonics is not significant for grid artifacts suppressing and may introduce a new artifact. Hence, we ignored the effect of harmonic components.

Finally, the two-dimensional IFFT is utilized to convert the frequency domain data into spatial domain data, and then we integrate the processed image blocks to reconstruct the image without grid artifacts. For a frequency block* G*(*u*,* v*) with size of* X×Y*, its two-dimensional IFFT is calculated as

(9) $$\begin{array}{c} f^{\prime }\left(\;x,y\;\right)=\overset{X-1}{\underset{u=0}{\sum }}\overset{Y-1}{\underset{v=0}{\sum }}G\left(\;u,v\;\right)e^{2\pi j\left(ux/X+vy/Y\right)} \end{array}$$

where *f*′(*x*,* y*) is the spatial domain data.

### 2.4. Data Acquisition

**Figure 3:**
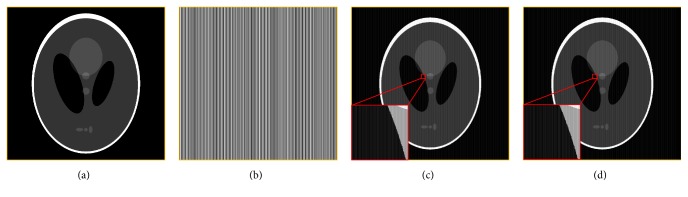
(a) Shepp-Logan phantom image; (b) the grid pattern of 3.5 lp/mm in spatial domain; (c) simulated image integrated with grid artifacts of 3.5 lp/mm; (d) simulated image integrated with grid artifacts of 3.49 lp/mm.

To verify the efficacy and efficiency of the proposed method, the proposed method is tested by a simulation study of the classic Shepp-Logan phantom and a real phantom study. For simulation data with grid artifacts acquisition, the simulated grid pattern is added to the Shepp-Logan phantom image. The Shepp-Logan phantom image with the size of 2048 × 2048 is shown in Figure 3(a) and the simulated grid pattern image with 3.5-line pair per millimeter (lp/mm) is shown in Figure 3(b). Figures 3(c) and 3(d) show the Shepp-Logan phantom images integrated with simulated grid artifacts of 3.49 lp/mm and 3.5 lp/mm, respectively.

**Figure 4:**
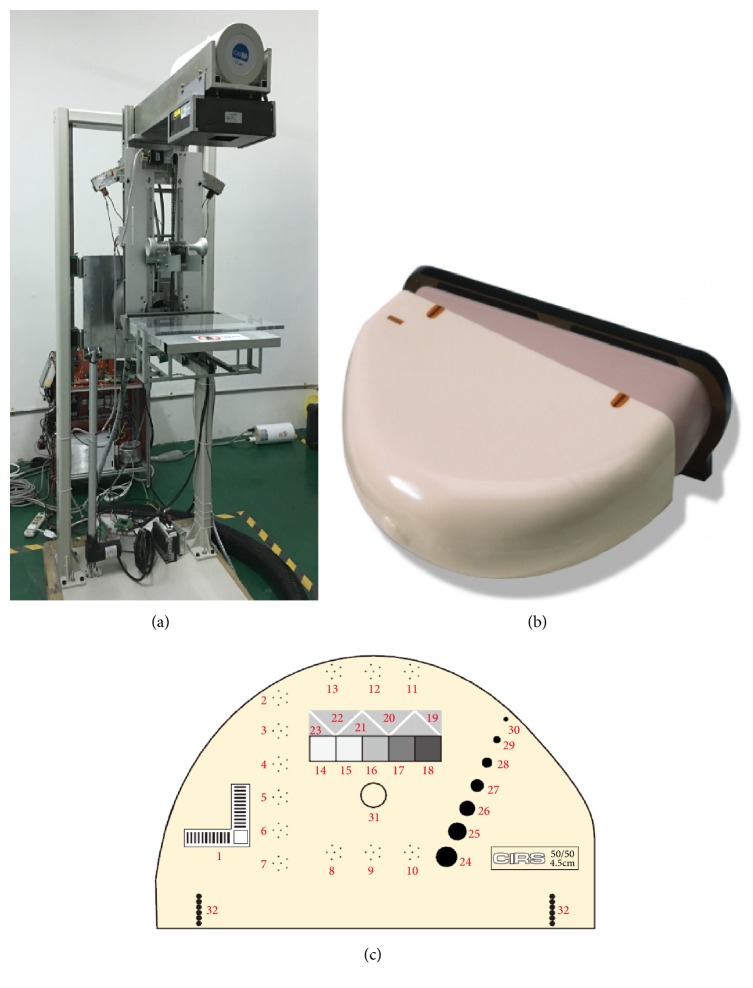
(a) Digital mammography system; (b) shape of CIRS 011A; (c) internal structure of CIRS 011A.

Furthermore, we performed a real phantom experiment with the digital mammography system as shown in Figure 4(a). The breast quality control phantom (CIRS, Inc., USA) [33] is used in this paper and its external and internal system structures are shown in Figures 4(b) and 4(c). In addition, the quality control phantom consists of 50% adipose material with 4.5cm thickness, 50% glands simulation material, and a removable 0.5cm equivalent layer of adipose tissue. In the study, an a-Se direct detector (AXS-2430, analogic Inc., Québec, Canada) with a pixel size of 0.085mm and the 2816 × 3584 resolution is employed.

**Figure 5:**
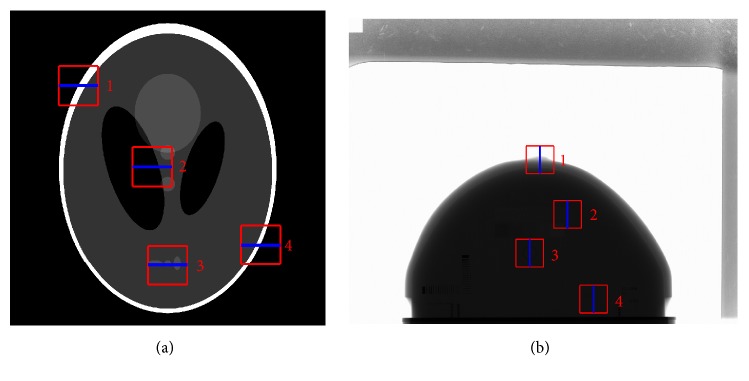
(a) Simulation image containing ROI#1, ROI #2, ROI #3, and ROI #4 in the red rectangles; (b) real image containing ROI#1, ROI #2, ROI #3, and ROI #4 in the red rectangles.

For qualitative evaluation in detail, we select four ROIs with central coordinates at (443, 487), (923, 1015), (1023, 1655), and (1627, 1523) in the simulation experiment, respectively.  Figure 5(a) shows ROI#1, ROI#2, ROI#3, and ROI#4 with size of 256 × 256 in the red rectangles, respectively. Concerning real image observation, we also select four ROIs with central coordinates at (1763, 1301), (2017, 1805), (1669, 2160), and (2257, 2584), respectively.  Figure 5(b) shows ROI#1, ROI#2, ROI#3, and ROI#4 with size of 256 × 256 in the red rectangles, respectively.

For the quantitative measurement, we utilize the normalized mean absolute distance (NMAB) to measure the difference between the conventional method and the proposed method. The NMAB of ROI is calculated:

(10) $$\begin{array}{c} NMAB=\frac{\sum_{i}^{M}\sum_{j}^{N}\left|f_{roc}\left(i,j\right)-f_{true}\left(i,j\right)\right|}{\sum_{i}^{M}\sum_{j}^{N}\left|f_{true}\left(i,j\right)\right|} \end{array}$$

where* f*_*roc*_(*i*,* j*) denotes pixel value at (*i*,* j*) in the corrected ROI,* f*_*true*_(*i, j*) represents pixel value at (*i*,* j)* in the reference ROI, and* M×N *is the size of ROI. Note that the smaller the NMAB, the closer the results between the original image and the corrected image.

For quality control phantom image, we propose an evaluation term named as mean value of specific direction (MVSD) to compare the difference between the conventional method and the proposed method. The MVSD of a pixel with the coordinate at (*i*,* j*) is shown as follows:

(11) $$\begin{array}{c} MVSD\left(\;i,j\;\right)=\frac{1}{N}\overset{N}{\underset{j}{\sum }}f_{roi}\left(\;i,j\;\right) \end{array}$$

where* f*_*roi*_(*i*,* j*) represents the pixel value at (*i, j*) in the ROI and *N* represents the width of ROI.

## 3. Results

### 3.1. Simulation Experiment

**Figure 6:**
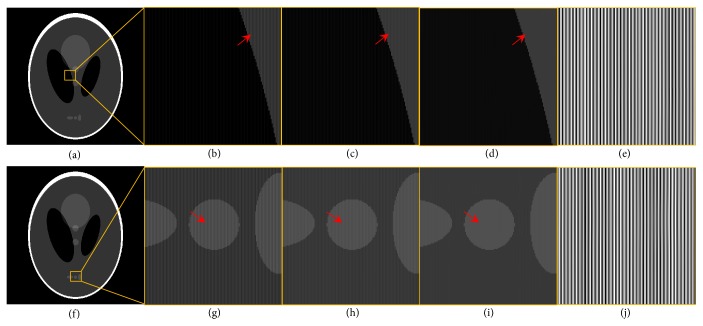
(a) Global image; (b)-(d) image without correction and images corrected by the global filter method and the proposed method of ROI#2, respectively; (g)-(i) image without correction and images corrected by the global filter method and the proposed method of ROI#4, respectively; ((e) and (j)) the spatial images of the difference between the images in (c), (h) and (d), (i) respectively; (f) global image.

In the simulated phantom experiment, we applied block processing with size 256 × 256 of image block, and the sizes of block and global filters are 15 × 15 and 51 × 51, respectively.  Figure 6 shows the simulated images with the grid artifacts of 3.49 lp/mm. Figures 6(b)–6(d) show the uncorrected image and the corrected images by the global filter method and the proposed method of ROI#2, respectively. Figures 6(g)–6(i) show the uncorrected image and the corrected images by the global filter method and the proposed method of ROI#4, respectively. As shown in the Figures 6(c) and 6(h), the global filter method can remove the grid artifacts to some extent. However, several grid artifacts are still present, which are indicated by the red arrows. Compared with global filter method, images corrected by the proposed method are visually better, as shown in Figures 6(d) and 6(i). Those grid artifacts indicated by the red arrows almost disappear in the corrected images by the proposed method. Figures 6(e) and 6(j) show the difference images by subtracting Figures 6(c) and 6(h) from Figures 6(d) and 6(i), respectively. Figures 6(e) and 6(j) show that the proposed method can suppress more grid artifacts than global filter method.

**Figure 7:**
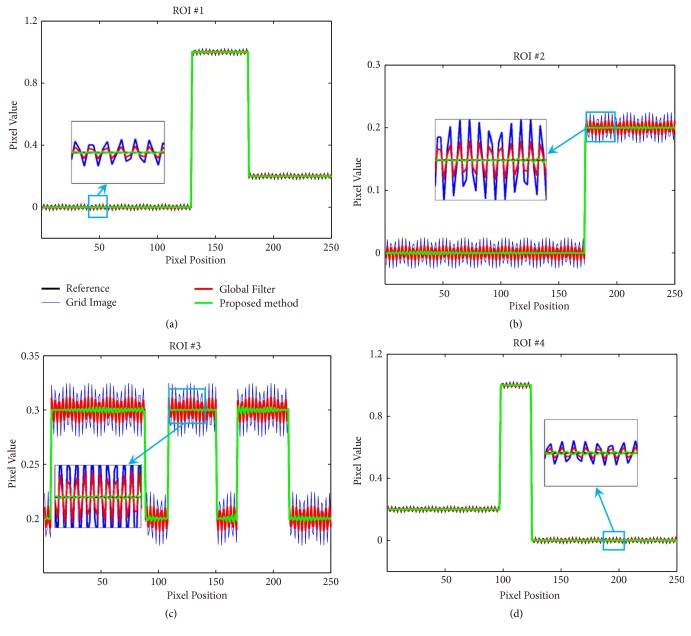
Image profiles indicated by the blue lines in ROI#1, ROI#2, ROI#3, and ROI#4 in Figure 5(a) with grid artifacts of the 3.49 lp/mm, respectively.

Figure 7 plots the horizontal profiles of blue lines in ROI#1, ROI#2, ROI#3, and ROI#4 in Figure 5(a), respectively. The profiles of the results obtained by the proposed method are much closer to the results of reference image than the results by the global filter method. As shown in the blue line, the image without any correction could not match the reference well because of the grid artifacts. This result partially proves that grid artifacts seriously degrade the quality of images. As shown in the red line, the global filter method could improve image quality to some extent. However, the fluctuation in the profile demands for further improvement. By contrast, the proposed method achieves high image quality, as shown in the green line profile.

The difference between the reference image and the corrected images by the global method and the proposed method is quantitatively evaluated by NMAB. The NMAB of ROI#1, ROI#2, ROI#3, and ROI#4 with the two grid modes are shown in Tables 1 and 2, respectively. Compared with the global filter method, the results of proposed method achieve an appreciable improvement, as shown in the last row of Tables 1 and 2.

### 3.2. Real Phantom Experiment

**Figure 8:**
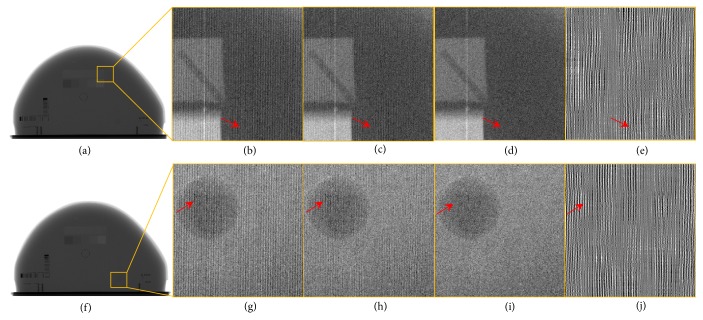
(a) Global image; (b)-(d) image without correction and images corrected by the global filter method and the proposed method of ROI#2, respectively; (g)-(i) image without correction and images corrected by the global filter method and the proposed method of ROI#3, respectively; ((e) and (j)) the spatial images of the difference between the images in (c), (h) and (d), (i) respectively; (f) global image.

In the real phantom experiment, we applied block processing with size 256 × 256 of image block, and the sizes of block and global filters are 11 × 11 and 61 × 61, respectively.  Figure 8 shows the images corrected with different methods in the real phantom experiment. Figures 8(b)–8(d) show the uncorrected image and the corrected images by the global filter method and the proposed method of ROI#2, respectively. Figures 8(g)–8(i) show the uncorrected image and the corrected images by the global filter method and the proposed method of ROI#3, respectively. As shown in Figures 8(c) and 8(h), the global filter method can remove the grid artifacts well. However, several grid artifacts are still present, which are indicated by the red arrows. Compared with the global filter method, the images corrected with the proposed method appear with fewer artifacts, as shown in Figures 8(d) and 8(i). The grid artifacts indicated by the red arrows almost entirely disappeared in the corrected images by the proposed method. Figures 8(e) and 8(j) display the difference images by subtracting Figures 8(c) and 8(h) from Figures 8(d) and 8(i), respectively. Figures 8(e) and 8(j) show that the proposed method can suppress more grid artifacts than the global filter method.

**Figure 9:**
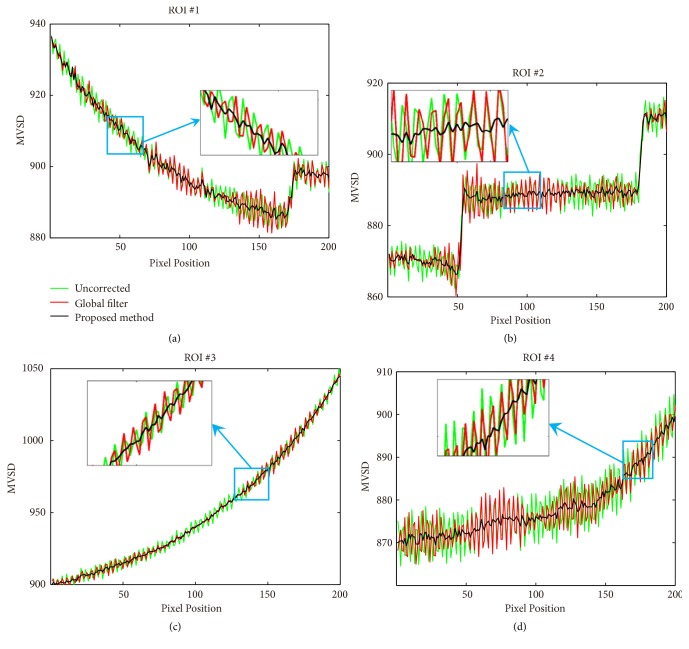
Image profiles indicated by the blue lines in ROI#1, ROI#2, ROI#3, and ROI#4 in Figure 5(b), respectively.

Figure 9 shows the vertical profiles of blue lines in ROI#1, ROI#2, ROI#3, and ROI#4 in Figure 5(b), respectively. The profiles of the results by the proposed method are much smoother than the results by the global filter method. As shown in the green line, the profile of the image without any correction shows a vibration with large amplitude because of the grid artifacts. This result partially proves that grid artifacts seriously degrade the quality of images. As shown in the red line, the utilization of the global filter method achieves the improvement of image quality. However, the fluctuation in the profile demands for further improvement. By contrast, the proposed method achieves high image quality and the fluctuation is relatively weak, as shown in the black line.

## 4. Discussion

In this paper, we propose a fast frequency signal filtering method based on image block processing. In the proposed method, image block processing is utilized to reduce the frequency interference between image blocks. Besides, we can employ multithread processing to decrease the computing time of CPU. In addition, characteristic frequency detection is employed in each block automatically to improve the fault-tolerance property of the grid accuracy. For optimal filtering, an improvement filter is constructed to minimize the influence of the artifacts filtering processing on the significant signals. The efficiency and applicability of the proposed algorithm are achieved by using simulated phantom data as well as real phantom data.

**Figure 10:**
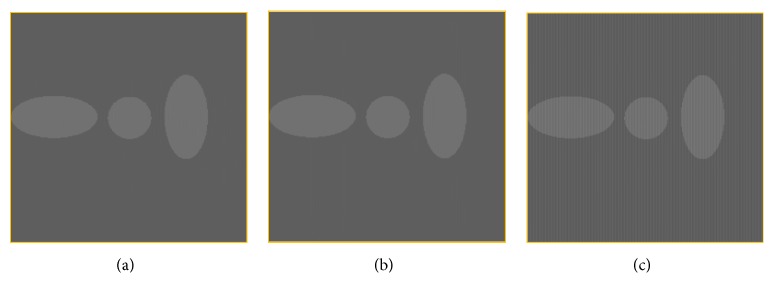
Images by block processing with the sizes of 128 × 128, 256 × 256 and 512 × 512, respectively.

There are several issues that we would like to discuss. Considering that the computational complexities for the global filtering are so serious for the current detector products, we combined block blocking and local filters to improve computation efficiency by using GPU multithread processing. In the real phantom experiment, the computation time is 2.278 s on a PC with i7(3.60 GHz) CPU and the time is 0.675 s by multithread processing on GPU (GTX 680) whose calculation efficiency has been increased by 3.4 times. For image block processing, Figure 10 shows the corrected images with different block size such as 128 × 128, 256 × 256, and 512 × 512. As shown in Figures 10(a) and 10(b), the block processing with sizes of 128 × 128 and 256 × 256 shows similar results, better than the result with size of 512 × 512 visually. And the time consuming is 0.680 s, 0.670 s, and 0.675 s, respectively. According to above-mentioned comparison, the optimal size of image block is 256 × 256. Additionally, we would also like to discuss the parameters *ς* and *σ*, which are closely related to detection range of characteristic frequency. By studying the frequency image, *ς* and *σ* can be determined at the appropriate frequency offset by experience and experiment. In the paper, the size of detection range is 7 × 7 at a 0.2 Hz frequency offset according to our needs.

**Figure 11:**
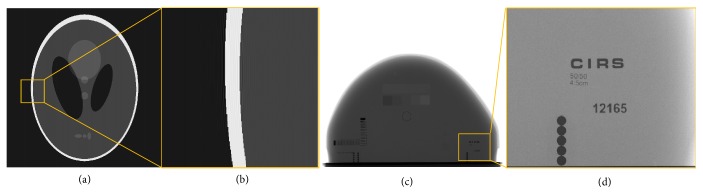
(a) Global simulation image by mean filter; (b) the corresponding magnified ROI of yellow squares in (a); (c) the real phantom image by mean filter; (d) the corresponding magnified ROI of yellow squares in (c).

The image quality may suffer damage more or less by filtering processing. As shown in formulas (7) and (8), the users can select the optimal filter size according to their needs. In our experiments, the filter size is 15 × 15 in the Shepp-Logan phantom experiment, and in the real phantom (CIRS. Inc., USA) experiment the filter size is 11 × 11. In frequency domain filtering, grid artifacts were removed by limiting the frequency components of grid. However, the loss of high frequency information could lead to ringing artifacts in most methods of grid artifacts suppressing. And ringing artifacts mainly exist near the contour edges of reconstructed images.  Figure 11 shows the corrected images by the mean filter. Figures 11(a) and 11(b) are global simulation image by mean filter and the corresponding magnified ROI of yellow squares in Figure 11(a). As shown in Figure 11(b), there are still a few ringing artifacts in simulated image due to the sharpness of gray value on the outline. However, in the real phantom experiment, these ringing artifacts almost disappeared visually as shown in Figure 11(d), which is the magnified ROI of yellow squares in Figure 11(c) by mean filter. And the filter results could basically be applied for clinical diagnosis. In the future, finding a better method to suppress ringing artifacts and grid artifacts will be the focus of our work.

## 5. Conclusion

In this study, the proposed integrated method, which has been tested in simulation system and the realistic systems, shows great potential for fast suppressing grid artifacts and generates high quality of digital mammography.

## Figures and Tables

**Table 1:** NMAB comparison of image with grid artifacts 3.49 lp/mm in different methods.

**NMAB**	**ROI#1**	**ROI#2**	**ROI#3**	**ROI#4**
**Uncorrected**	0.0630	0.1799	0.0738	0.0880
**Global Filter**	0.0290	0.0824	0.0344	0.0405
**Proposed Method**	0.0047	0.0098	0.0040	0.0063

**Table 2:** NMAB comparison of image with grid artifacts 3.5 lp/mm in different methods.

**NMAB**	**ROI#1**	**ROI#2**	**ROI#3**	**ROI#4**
**Uncorrected**	0.0630	0.1799	0.0738	0.0880
**Global Filter**	0.013	0.027	0.008	0.025
**Proposed Method**	0.0047	0.0095	0.0040	0.0062
